# Meteorological Observations

**Published:** 1860-04

**Authors:** J. G. Westmoreland

**Affiliations:** The Smithsonian Institution, Jan., 1860, at Atlanta, Ga.


### METEOROLOGICAL OBSERVATIONS,

Taken by J. G. Westmoreland, M. D., for the Smithsonian Institution, Jan., 1860, at Atlanta, Ga., Latitude 33 deg. 45 min.—Longitude 7 deg. 30 min.—Height above the Sea, 1050 feet.

| Days of Month | BAROMETER. | | | THERMOMETER. | | | RAIN AND SNOW. | | CLOUDS. | | | WIND. | | |
|---|---|---|---|---|---|---|---|---|---|---|---|---|---|---|
| | 7 A.M. | 2 P.M. | 9 P.M. | 7 A.M. | 2 P.M. | 9 P.M. | Amt. Rn | In. Sn'w | 7 A.M. | 2 P.M. | 9 P.M. | 7 A.M. | 2 P.M. | 9 P.M. |
| 1 | 30.10 | 30.15 | 30.15 | 46 | 62 | 44 | | | 0 | 0 | 0 | W | W | W |
| 2 | 30.15 | 30.25 | 30.20 | 40 | 68 | 50 | | | 0 | 0 | 0 | W | W | W |
| 3 | 30.20 | 30.25 | 30.20 | 48 | 68 | 60 | | | 5 | 0 | 5 | S E | S E | S E |
| 4 | 30.30 | 30.25 | 30.20 | 50 | 64 | 58 | | | 0 | 5 | 5 | W | W | W |
| 5 | 30.10 | 30.05 | 30.05 | 48 | 56 | 48 | 0.25 | | 10 | 5 | 10 | E | S W | S W |
| 6 | 30.00 | 30.05 | 30.00 | 48 | 76 | 58 | | | 0 | 0 | 0 | W | W | W |
| 7 | 30.00 | 30.05 | 30.00 | 60 | 76 | 68 | | | 0 | 0 | 0 | W | W | W |
| 8 | 30.00 | 29.85 | 29.80 | 62 | 74 | 70 | 0.12 | | 0 | 5 | 10 | S W | S W | S W |
| 9 | 29.95 | 29.95 | 29.95 | 34 | 50 | 40 | | | 0 | 0 | 0 | N W | N W | N W |
| 10 | 29.90 | 29.95 | 30.00 | 36 | 54 | 40 | | | 0 | 0 | 0 | N W | N W | N W |
| 11 | 30.10 | 30.15 | 30.00 | 32 | 52 | 48 | | | 0 | 0 | 0 | N W | N W | N W |
| 12 | 29.90 | 29.85 | 29.95 | 42 | 62 | 34 | | | 5 | 5 | 5 | S W | W | W |
| 13 | 30.00 | 30.05 | 30.00 | 30 | 50 | 40 | | | 0 | 0 | 0 | N W | N W | N W |
| 14 | 29.90 | 30.00 | 30.00 | 34 | 52 | 48 | | | 0 | 0 | 0 | N W | N W | N W |
| 15 | 30.00 | 30.00 | 30.05 | 40 | 60 | 54 | | | 0 | 0 | 5 | N W | W | S W |
| 16 | 30.10 | 30.05 | 30.00 | 46 | 50 | 44 | | | 5 | 10 | 10 | S | S E | S E |
| 17 | 30.00 | 29.95 | 29.90 | 48 | 52 | 46 | | | 10 | 10 | 10 | E | E | E |
| 18 | 29.90 | 29.90 | 29.90 | 48 | 56 | 50 | | | 10 | 10 | 10 | E | E | E |
| 19 | 29.98 | 30.00 | 30.00 | 50 | 63 | 55 | | | 10 | 5 | 0 | E | N E | N |
| 20 | 30.05 | 30.10 | 30.03 | 50 | 58 | 48 | | | 0 | 0 | 0 | N W | W | N |
| 21 | 30.05 | 30.15 | 30.10 | 38 | 56 | 40 | | | 0 | 0 | 0 | N | N W | N W |
| 22 | 30.08 | 30.12 | 30.05 | 40 | 68 | 58 | | | 0 | 0 | 0 | W | W | W |
| 23 | 30.00 | 30.00 | 29.90 | 48 | 66 | 56 | 0.38 | | 5 | 10 | 10 | S E | S E | S E |
| 24 | 29.75 | 29.80 | 29.80 | 46 | 56 | 38 | | | 5 | 0 | 0 | N W | N W | N W |
| 25 | 29.80 | 29.85 | 29.90 | 34 | 48 | 36 | | | 0 | 0 | 0 | N W | N W | N W |
| 26 | 29.95 | 30.00 | 30.00 | 34 | 50 | 38 | | | 0 | 0 | 0 | N W | N W | N W |
| 27 | 30.05 | 30.05 | 30.00 | 34 | 48 | 38 | | | 0 | 0 | 0 | N W | N W | N W |
| 28 | 29.90 | 29.95 | 29.90 | 36 | 56 | 46 | | | 0 | 0 | 0 | N W | N W | N W |
| 29 | 29.90 | 30.00 | 30.00 | 40 | 60 | 50 | | | 0 | 0 | 0 | N W | N W | N W |
| 30 | 29.90 | 29.95 | 29.95 | 42 | 64 | 48 | | | 0 | 0 | 0 | W | W | W |
| 31 | 29.95 | 29.80 | 29.90 | 48 | 68 | 60 | | | 0 | 0 | 10 | W | W | S |

EXPLANATION OF TABLE.—0 Signifies Entire Clearness—5 Half Cloudy—10 Entire Clearness.

